# Precision Medicine Gains Momentum: Novel 3D Models and Stem Cell-Based Approaches in Head and Neck Cancer

**DOI:** 10.3389/fcell.2021.666515

**Published:** 2021-07-08

**Authors:** Annette Affolter, Anne Lammert, Johann Kern, Claudia Scherl, Nicole Rotter

**Affiliations:** Department of Otorhinolaryngology, Head and Neck Surgery, University Hospital Mannheim, Medical Faculty Mannheim, Heidelberg University, Mannheim, Germany

**Keywords:** head and neck cancer, 3D cell culture, cancer stem cell models, bioprinting, personalized therapy

## Abstract

Despite the current progress in the development of new concepts of precision medicine for head and neck squamous cell carcinoma (HNSCC), in particular targeted therapies and immune checkpoint inhibition (CPI), overall survival rates have not improved during the last decades. This is, on the one hand, caused by the fact that a significant number of patients presents with late stage disease at the time of diagnosis, on the other hand HNSCC frequently develop therapeutic resistance. Distinct intratumoral and intertumoral heterogeneity is one of the strongest features in HNSCC and has hindered both the identification of specific biomarkers and the establishment of targeted therapies for this disease so far. To date, there is a paucity of reliable preclinical models, particularly those that can predict responses to immune CPI, as these models require an intact tumor microenvironment (TME). The “ideal” preclinical cancer model is supposed to take both the TME as well as tumor heterogeneity into account. Although HNSCC patients are frequently studied in clinical trials, there is a lack of reliable prognostic biomarkers allowing a better stratification of individuals who might benefit from new concepts of targeted or immunotherapeutic strategies. Emerging evidence indicates that cancer stem cells (CSCs) are highly tumorigenic. Through the process of stemness, epithelial cells acquire an invasive phenotype contributing to metastasis and recurrence. Specific markers for CSC such as CD133 and CD44 expression and ALDH activity help to identify CSC in HNSCC. For the majority of patients, allocation of treatment regimens is simply based on histological diagnosis and on tumor location and disease staging (clinical risk assessments) rather than on specific or individual tumor biology. Hence there is an urgent need for tools to stratify HNSCC patients and pave the way for personalized therapeutic options. This work reviews the current literature on novel approaches in implementing three-dimensional (3D) HNSCC *in vitro* and *in vivo* tumor models in the clinical daily routine. Stem-cell based assays will be particularly discussed. Those models are highly anticipated to serve as a preclinical prediction platform for the evaluation of stable biomarkers and for therapeutic efficacy testing.

## Conventional Treatment Strategies and New Therapeutic Concepts in Head and Neck Cancer

Head and neck squamous cell carcinoma (HNSCC) is a heterogeneous tumor entity with varying clinical presentation and prognosis. Current treatment options for the disease have limited success particularly in recurrent or metastatic stage. For early stage disease, surgery and radiotherapy (RT) are the main pillars. Concurrent platinum-based chemotherapy with irradiation is standard-of-care for locoregionally advanced tumors, both in an adjuvant or primary setting. However, especially the present non-surgical standard-of-care treatment with concurrent high-dose cisplatin (100 mg/m^2^) every 3 weeks and RT (70 Gy over 6–7 weeks) entails severe acute and late toxicities (Denis et al., 2003) as well as functional deficits resulting in a reduced quality of life. For patients who are not eligible for cisplatin-based chemotherapy, the anti-epidermal growth factor receptor (EGFR) monoclonal antibody cetuximab is an option and can be administered in combination with RT. So far, cetuximab is the only EMA-approved targeted therapy agent for HNSCC. In the case of recurrent or metastatic (R/M) disease, platinum-based chemotherapy is the mainstay of therapy, and the addition of cetuximab to standard cytotoxic chemotherapy offers some benefit in the first-line setting, but at the cost of increased toxic effects. Unfortunately, there is no biomarker currently available to be able to predict whether HNSCC patients will benefit from EGFR blockade or not (Egloff and Grandis, 2009). A new combination of cetuximab with another agent that has been recently presented is the taxane-based TPEx scheme which has been demonstrated to exert lower toxicity along with a reduced duration of therapy (Guigay et al., 2019) in a palliative setting.

Over a long period, there was no standard second-line therapy for advanced R/M HNSCC after platinum-based chemotherapy. Two immune checkpoint inhibitors (CPI), nivolumab, and pembrolizumab targeting programmed cell death protein 1 recently got approval for mono- or combination therapy (Ferris et al., 2016; Burtness et al., 2019). Immune CPI are amongst the central developments in oncology in the last decade, but the response is very heterogeneous for many entities, including HNSCC. However, despite encouraging results, the overall response rates of these agents only range from 13 to 18% (Ferris et al., 2016; Larkins et al., 2017). Primary resistance against CPI is seen in up to 60% of all patients, including HNSCC (Topalian et al., 2012).

The combination of immune CPI with established therapies (irradiation, chemotherapy, and cetuximab) is currently being tested in several clinical studies (Seliger, 2019), however up to now the effect of such conventional treatments on immune checkpoints remains unclear. Considering that serious immune-associated toxicity can occur during immunotherapy, it is absolutely necessary to identify predictive biomarkers to predict the response of the tumor to checkpoint blockade (Bai et al., 2020). In addition to the immunohistochemical evaluation of the presence of PD-L1, immune cell infiltration, the mutation load of the tumor or the expression profile of immune-associated genes are currently being investigated (Partlová et al., 2015; Mandal et al., 2016; Chen X. et al., 2018; Hanna et al., 2018; Oliva et al., 2019; Wang et al., 2019). However, no valid predictive markers for HNSCC have been established so far.

Newly developed technologies, such as high-throughput sequencing might be essential to determine differences in genetic, epigenetic, biological, and immunological properties of HNSCC. Furthermore, etiological risk factors such as tobacco and alcohol abuse as well as infection with human papillomavirus (HPV) have a great impact on the treatment outcome. The rapid increase in the incidence of HPV-positive oropharyngeal cancer in younger patients and the more favorable prognosis observed in this subgroup (Ang et al., 2010) have led to the concept of treatment de-escalation for HPV-driven cancers, as recently reviewed by Mehanna et al. (2020). Several de-escalation studies worldwide have been initiated over the past decade. Strategies include a dose reduction of RT as well as decreasing dosages of systemic therapy or omitting platinum based chemotherapy (Mehanna et al., 2020). These studies aimed to improve quality of life by lowering toxic side effects with the same probability of survival. It is important to notice, that the biological basis for de-escalation remains unclear. Also the first trials DE-ESCALATE and RTOG1016 did not identify successful de-escalation strategies.

Some studies report an improvement to salvage chemotherapy (SCT) after exposure to immune CPI for different tumor entities that exposure to ICI improves response to SCT (Schvartsman et al., 2017; Rossi et al., 2018; Szabados et al., 2018). Saleh et al. (2019) confirm this finding for HNSCC and an increased response rate to chemotherapy (30%) administered after progression on ICI in patients with R/M SCCHN. However, this finding has to be confirmed in additional cohorts and prospective clinical trials and needs to be optimized. The prognosis for HNSCC patients who progress on CPI is dismal, a situation providing the rationale for the identification of new (targeted) treatments.

## Value of Precision Medicine: Current State of Predictive Markers

The future of cancer therapy may lie in treatments designed precisely for a specific type of cancer. Individualized targeted therapies might inhibit local and distant tumor growth by interfering with the molecular pathways that cause cancer cells to proliferate and survive.

The term “precision medicine” refers to different approaches: targeted therapy, immunotherapy, or genomics. However, all these concepts have the aim to align medical care with molecular and possibly also environmental and lifestyle factors of certain patient groups. Optimizing the success rates of modern therapies with less adverse effects is the main aim in precision medicine. Biomarker-supported therapy is an essential sub-discipline of precision medicine. Biomarkers are DNA-, mRNA-, or protein-based and comprise driver mutations, protein expression, mRNA, MSI (microsatellite instability), tumor mutational burden (TMB), and epigenetic biomarkers, e.g., specific methylation patterns of DNA.

The only targeted therapy approved in R/M HNSCC is the antibody against EGFR, cetuximab (Licitra et al., 2011, 2013), pembrolizumab (Seiwert et al., 2016), and nivolumab (Ferris et al., 2016) targeting PD-1 examples for immune-CPI. Remarkably, the decision to administer these therapeutic compounds is not biomarker-based (Malone and Siu, 2018).

Although EGFR overexpression is observed in > 90% of HNSCC (Dassonville et al., 1993; Rubin Grandis et al., 1996) and is associated with an unfavorable clinical outcome (Rubin Grandis et al., 1998; Ang et al., 2004) the correlation with response to treatment is inconsistent (Vermorken et al., 2007). So far, no molecular marker has been identified to correlate with HNSCC response to EGFR-targeting in patients. Tumor EGFR copy number did not turn out as a predictive biomarker for the efficacy of cetuximab plus platinum/5-FU as first-line therapy for patients with R/M HNSCC (Licitra et al., 2011).

Immunotherapeutic anti-PD-1 agents have achieved to become standard-of-care for platinum-refractory R/M HNSCC as they have proven to show evidence of survival benefit and long-term responses. However, appropriate stratification of patients who will benefit from immunotherapy is crucial due to response rates below 20% (Ferris et al., 2016; Larkins et al., 2017). Research is currently focusing on the identification of CPI response predictors. The issue whether PD-L1 expression is a reliable biomarker of response in HNSCC has been recently addressed in different studies. There is a general tendency of PD-L1 expressing tumors to show superior response rates to CPI compared to PD-L1 negative tumors (Hansen and Siu, 2016). This observation has been endorsed by the KEYNOTE-040 and -048 trials in R/M HNSCC where survival in PD-L1 expressing cases was significantly increased (Burtness et al., 2019; Cohen et al., 2019). Interestingly, no significant association between PD-L1 levels and response to nivolumab or survival were found in the CHECKMATE-141 trial (Ferris et al., 2016, 2018). These diverging findings might be due to differences in the assays when determining the PD-L1 status on the one hand. On the other hand, there is evidence that PD-L1 is regulated by multiple signaling pathways that are frequently altered and known as survival pathways in HNSCC such as PI3K/Akt, or MAPK (Lui et al., 2013; Lawrence et al., 2015).

These molecular interactions and furthermore their varying expression over time between first diagnosis and progression, metastases or recurrence, after acquiring therapy resistance or during treatment have demonstrated the dynamics of PD-L1 in multiple tumor entities (Gadiot et al., 2011; Darb-Esfahani et al., 2016; Han et al., 2016; Ock et al., 2017). It is worth mentioning that, in a smaller percentage, PD-L1 negative tumors also respond to CPI. The success of immunotherapy is guided by various other factors such as association with HPV, the infiltration of the tumor with immune cells, or TMB. So far, it has been demonstrated that response to immune CPI is correlated with increased TMB. This might imply that prior therapy with DNA damaging compounds may enhance sensitivity to CPI due to increased TMB. In HNSCC indeed one study demonstrated that prior treatment with chemotherapy was associated with increased overall survival relative to patients with prior surgery or radiation therapy in an observational study of patients with head and neck squamous cell carcinoma (HNSCC) treated with anti-programmed cell death 1 ligand 1 (anti-PD-L1) (Hanna et al., 2018). Yet, this thesis was not substantiated in other cancer entities (Pulte and Brenner, 2010; Reck et al., 2016; Forde et al., 2018) or further studies. It has to be taken into account that prior chemotherapy may as well enhance subclonal mutations and intratumoral heterogeneity, features that are discussed to negatively correlate with sensitivity to CPI.

Currently, additional biomarkers of response to anti-PD-1/PD-L1 agents in HNSCC have been proposed. The oral and intestinal microbiota is discussed as a candidate marker as this ecosystem correlates microenvironment and tumor microenvironment (TME) as they may regulate environmentally induced immune responses and ultimately impact on therapeutic efficacy Its composition has not only shown an impact on PD-L1 efficacy but is also correlated with response to treatment in different cancers. This is in line with studies suggesting an association with other tumoral features such as progression or recurrence (Oliva et al., 2019).

Recently published studies on patients with HNSCC indicate that the level of PD-1 expression by CD8 + T cells is associated with cell functionality and overall survival of the patient. Kansy et al. found a higher frequency of PD-1 expression that was upregulated on TIL in HPV-positive patients with a significantly better clinical outcome. In a murine HPV+ model treated with anti-PD-1 mAb where PD-1^*high*^/^*low*^ populations were differentially modified. Different PD-1 expression levels lead to the interpretation of PD-1 expression as a marker of competent tumor reactive T cells while PD-1^*high*^ expression was interpreted as an indicator of exhaustion of dysfunctional cells negatively impacting on the TME. For validation, baseline PD-1 levels need to be correlated with patient responder status (Kansy et al., 2017).

Why are there currently no validated biomarkers predicting response that are comprehensively applicable to all HNSCC patients? Oliva et al. explain this issue by the fact that most investigations on HNSCC biomarkers have been performed retrospectively by using baseline archival tumor material, which does not mirror spatial and tumoral heterogeneity. They claim that it is not sufficient to separately evaluate potential predictors. To take account of the complexity of immune responses, markers should always be analyzed in the context with other factors, and interactions, especially between the immune system and the TME, should be thoroughly considered (Oliva et al., 2019).

## Environmental and Life-Style Determinants of HNSCC

For disease prevention or control, the recognition of main social and behavioral variables and implementation into appropriate programs and policies is mandatory. Addressing of these variables would reduce the risk of serious diseases such as cancer thereby improving popular health (Allam and Windsor, 2013). In HNSCC, most approaches refer to oral cancer. Tobacco and alcohol usage, tobacco chewing and dietary malnutrition are the most important downstream social determinants (Llewellyn et al., 2001). Hobdell et al. (2003) published an association between socioeconomic status (SES) variables and oral health. They observed a distinct gradient between the most highly and least socio-economically developed countries and the incidence of oral diseases including cancer, dental caries, and destructive periodontal disease. Attributable risk factors also comprise diet deficiencies. Fresh food contains antioxidants and anti-carcinogenic agents which might help oppose the damaging influence of carcinogens such as smoking, alcohol drinking or tobacco chewing (Bosetti et al., 2003; Boccia et al., 2008). Employment in certain sectors can enhance the risk for oral malignancies i.e., by exposure to formaldehyde, or by working in painting and printing, textile and electronic factory jobs (Allam and Windsor, 2013). Vučičević Boras et al. compared the environmental and behavioral risk factors living environment, occupational exposure, education, residence, family cancer, diet, smoking, and alcohol consumption parameters in patients with head and neck cancer (HNC) with a control group. They discussed smoking and low education as significant risk factors for HNC regardless of gender. Family HNC and breast cancer were significant risk predictors (Vučičević Boras et al., 2019). Omics-based approaches might offer novel tools for diagnosis and treatment of head and neck malignancies in the field of precision health (Adeola et al., 2019). Omics technologies comprehensively screen for early changes in DNA, RNA, protein, and metabolite expression (Rai et al., 2018) and may contribute to the clearly needed early detection of oral cancer. Disruption of the circadian clock was recently linked to head and neck pathologies, such as oral cancer and Sjögren syndrome (Matsumoto et al., 2016; Adeola et al., 2019). Nearly half of all protein encoding genes are subject to circadian rhythms in transcription, mostly organ-unspecifically (Zhang et al., 2014). Hence, circadian variations in multi-omics analyses, recently called circadiOmics are discussed as a relevant step toward unbiased precision health (Ceglia et al., 2018).

## Cancer Stem Cell Markers as Prognosticators in HNSCC

In solid tumors, in addition to the main tumor mass consisting of well-differentiated cells, a subpopulation of immature tumor cells called cancer stem cells (CSCs) exists. CSC show unlimited proliferative capacity, have the ability for self-renewal, differentiation, and tumor invasion, and are capable of DNA damage repair. CSC-related factors as well as the TME both contribute to radioresistance and reveal new therapeutic approaches (Albers et al., 2012; Arnold et al., 2020).

CD44+, a cell membrane-bound glycoprotein that occurs in several isoforms, is considered as a marker for the CSC phenotype (Zhang et al., 2012). These isoforms are generated by alternative splicing of a region of variable exons. They differ in their amino acid sequence and their amount of N- and O-glycosylation (Franzmann et al., 2007), whereby their apparent molecular weight varies between 85 and 250 kilo Dalton (kD) (Saito et al., 1998). At least 20 variants of CD44 have been reported. They arise through alternative splicing of 10 exons, which encode the proximal part of the respective extracellular domain (Screaton et al., 1993; Ponta et al., 1998). For the first time, CD44 was described as a receptor on circulating lymphocytes, where it conveys homing, cell adherence and migration (Stamenkovic et al., 1989). Prince et al. (2007) showed that CD44+ tumor cells, which typically make up < 10% of all HNSCC cells, were able to develop a new primary *in vivo*, while CD44 – cells were not. Günthert et al. (1991) demonstrated that the expression of CD44 caused metastatic potential in a non-metastatic cell line in the rat model. Since then, various analyses have been initiated that imply the correlation between CD44 expression and tumor progression, metastasis, and prognosis. Such associations exist in several epithelial tumor entities, besides HNSCC in colorectal carcinoma (Thenappan et al., 2009), breast carcinoma (Park et al., 2010), and different types of gastric carcinoma (Okayama et al., 2009).

Previously we observed CD44 in human HNSCC tumor tissue samples by immunofluorescence (Faber et al., 2011). Other research groups used flow cytometry (Prince et al., 2007), immunohistochemistry or microarray technology (Han et al., 2009) to verify CD44+ cells in HNSCC. The results consistently postulate the presence of CD44 in HNSCC tumors at both protein and gene levels. Prince et al. (2007) published that CD44+ tumor cells usually make up < 10% of all cells in the entirety of HNSCC. The percentage of CD44+ cells within HNSCC is subject to inter-individual fluctuations. Here, the proportion of CD44+ cells varied from 4% to over 90% (Pries et al., 2008). A possible explanation for these extreme variations is the fact that different methods (FACS analyzes versus immunohistochemistry) have been used in these studies.

We and others (Herold-Mende et al., 1996; Han et al., 2009) described a surface staining pattern of CD44 in HNSCC cell lines and tissue samples (Faber et al., 2011), an observation that indicates its role as an adhesion molecule during tumor survival and progression. CD44 is supposed to attach the cells to the extracellular matrix (ECM). The adherence of malignant cells must be alternated in order to be able to detach from the primary tumor and to form metastases elsewhere (Schirrmacher, 1985). In various tumor cell lines and human tumors the extracellular portion of CD44 serves as a substrate for proteolytic cleavage processes by metalloproteinases (MMPs) (Okamoto et al., 2002). Remarkably, CD44 expression could be found mainly in the area of the tumor invasive front, which is in direct contact with the stromal cells surrounding the tumor and forms the tumor stem cell niche in HNSCC (Faber et al., 2013a).

Already in 2010, Joshua et al. suggested that flow cytometric measurements of the frequency of Lin-CD44þ cells may provide a prognostic test for patients with HNSCC. They observed a correlation between a high frequency of Lin-CD44þ cells with tumor aggressiveness represented by factors such as advanced T classification and recurrence (Joshua et al., 2012). Chen et al. (2014) suggested that CD44 is related to worse T category, N category, tumor grade and prognosis in pharyngeal and laryngeal cancer, but no clear association was revealed between CD44 expression and oral cancer. Jakob et al. (2020) recommend testing for CSC markers in patients with advanced or late stage HNSCC, as they observed correlations between CSC markers ALDH1, BCL11B, BMI-1, and CD44 and prognosis.

ALDH1 is a human aldehyde dehydrogenase that can be used to identify both physiological stem cells and CSC (Ma and Allan, 2011). The expression of ALDH1 is associated with an increased incidence of metastases (Kim et al., 2015). In contrast, ALDH1 is associated with tumor malignancy and cell self-renewal potential in head and neck tumors, but there is no significant correlation with the 5-year survival rate of the patients examined. Although ALDH1 has been shown to play a role in the maintenance of CSC in HNSCC, there is no correlation whatsoever with the occurrence of lymph node metastases. Furthermore, stem cell properties in HNSCC were interrelated with other surface markers such as Sox2 and OCT3 for the first time (Huang et al., 2014; Yu and Cirillo, 2020). ALDH1 is associated with squamous cell carcinomas in other locations than head and neck. The marker was primarily found in the invasive front and in metastatic lesions of esophageal carcinomas (Yang et al., 2014) exhibiting a more aggressive potential for invasion and metastasis.

In HNSCC, ALDH1 expression is not relevant for therapeutic decisions, although it was shown to be associated lymph node metastases (Michifuri et al., 2012; Yu and Cirillo, 2020). A meta-analysis by Dong et al. from 2017 summarized 14 studies with a total of 1258 patients regarding the effect of ALDH1 in HNSCC. They demonstrated a significant correlation of ALDH1 with tumor differentiation and reduced overall survival (Dong et al., 2017). According to Leinung et al. CD44 is found more ubiquitously in HNSCC compared to ALDH1. As a result, ALDH1 seems to be more suitable to identify a certain CSC subpopulation. However, the authors state that neither CD44 nor ALDH1 alone or in combination is suitable to detect CSC separately in HNSCC (Leinung et al., 2015). Besides CD44 and ALDH1, several other stem cell markers have been described for HNSCC, such as Bmi-1, CD133, Nanog, Oct-4, and SOX2 (Satpute et al., 2013; Patel et al., 2014; Qian et al., 2015) and those have partly been associated with prognosis (Chen et al., 2014; Dong et al., 2014; Zhou and Sun, 2014). However, as methodology, patient cohorts, and sample quality in studies are not standardized yet, the role of stem cell markers in HNSCC remains unclear and the prognostic value is discussed controversially (Fan et al., 2017). Some studies link CD133 to lymph node metastases in HNSCC (Mannelli et al., 2015). However, this observation was based only on a small HNSCC sample number. If detectable, CD133 seems to be associated with a tendency to metastasize (Tang et al., 2013; Mannelli et al., 2015).

Some groups have recently suggested further molecular stem cell markers in squamous cell carcinoma, including SOX2, where, in particular, a co-expression of ALDH1 and SOX2 was found (Huang et al., 2014). The SOX2 gene encodes for a transcription factor that is responsible for maintaining the self-renewal capacity in physiological stem cells and neural progenitor cells (Adachi et al., 2010). It has only recently been associated with stem cell properties in malignant cells (Huang et al., 2014).

In summary, it can be stated that there is no single tumor stem cell marker and no combination of markers currently established in HNSCC. Features of malignancy, such as susceptibility to metastasis and recurrence were related to varying expression levels of various stem cell markers.

Immune CPI are one of the central developments in oncology during the last decade. Unfortunately, as has been mentioned above the response is very heterogeneous in many tumor entities, including HNSCC. The efficacy of CPI is limited by the capacity of tumor cells to escape the immune system. CSCs are supposed to play a crucial role in this process as they are known to contribute to the formation of metastases and recurrences. However, the informative value of stem cell markers as predictors may be limited due to the distinct intratumoral heterogeneity as well as tumor/metastasis heterogeneity in HNSCC. Expression levels may be subject to variability within the tumor and during tumor progression (Ihler et al., 2018). To be able to take all these aspects into consideration, innovative preclinical tumor models need to be established and standardized.

## Environmental and Life-Style Influences in CSC Behavior

As many other cell types, CSC are also regulated by a variety of extrinsic microenvironmental stimuli and adapt to changing environmental conditions such as hypoxia or nutrient deficiency (Peitzsch et al., 2019a,b). Under the exposure of specific stimuli, stem cells are capable of acquiring a specific phenotype. Stem cell behavior can be influenced by various factors such as oxygen concentrations. Adult stem cells have the ability to shift toward an oxidative metabolism once remain in a state of quiescence in their specialized niche until external signals induce a metabolic shift toward an oxidative metabolism (Kumar et al., 2017).

One critical extrinsic factor is nicotine. Yu et al. suggest by their data that this noxious agent may play a critical role in the development of tobacco-induced cancers by regulating CSC features, and that these effects are likely mediated through pathways that promote epithelial-mesenchymal transition (EMT). Nicotine promotes the CSC phenotype thereby enhancing the tumor-propagating capability of HNSCC cells (Yu et al., 2012). In NSCLC cells it has also been shown that nicotine can induce expression of Sox2 as well as mesenchymal markers and enhance migration and stemness (Schaal et al., 2018). Alcohol has also been proposed as another key factor for the development of HNSCC as there is evidence that alcohol increases CSC population, thereby promoting aggressiveness, recurrence, and therapy resistance of cancers.

Activation of signaling compounds such as MAPK, Wnt/GSK3β/β-catenin, and TLR4/Nanog, and alterations of the TME induced by alcohol cause the promotion of CSC. These environmental factors, which frequently apply to HNSCC patients may reveal novel therapeutic approaches targeting the respective multi-components/cascades regulating characteristics of CSC (Xu and Luo, 2017). Based on the results from preclinical *in vitro* and *in vivo* studies it is likely that combining chemotherapy with CSC-targeting agents may help to overcome resistance against conventional chemotherapy. A variety of compounds targeting CSC differentiation and cell death cascades in combination with chemotherapy are currently being investigated in clinical trials (Li et al., 2017). After completion, new insights about safety and efficacy of these combination schemes are expected.

## Preclinical Tumor Models for HNSCC

**FIGURE 1:**
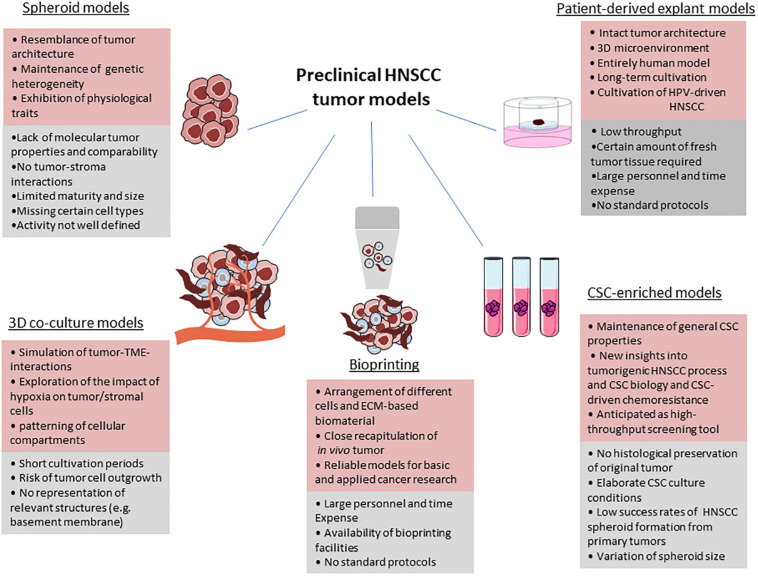
Benefits, limitations and drawbacks of current preclinical HNSCC models. For each model, advantages (red), and limitations (grey) are given. Parts of the figure were drawn by using pictures from Servier Medical Art (http://smart.servier.com/), licensed under a Creative Commons Attribution 3.0 Unported License (https://creativecommons.org/licenses/by/3.0/).

Preclinical models that precisely predict clinical outcomes are urgently needed in the field of cancer drug discovery and development (Figure 1).

Under optimal conditions, tumor tissue can be kept in culture over a period of sufficient length to be able to investigate short- and long-term effects of therapy. By testing different treatment strategies, the outcome of the individual patient can be analyzed before starting treatment and will help assign patients to their optimal therapy. Due to the existent progression in the development of new therapy regimens such as targeted therapies and immunotherapies, cell culture techniques also attained increasing prominence, in particular those that grow in three-dimensional (3D) architecture (Demers et al., 2020) (Table 1).

**TABLE 1:** Preclinical 3D models for HNSCC.

Tumor Derivation	HNSCC
Spheroids	Köpf-Maier and Zimmermann, 1991
	Eke et al., 2009
	Storch et al., 2010
	Eke et al., 2013
	Hagemann et al., 2017
	Hagemann et al., 2018
	Melissaridou et al., 2019
	Driehuis et al., 2019

Co-cultures	Hoffmann et al., 2009
	Sawant et al., 2016
	Young et al., 2018
	Huang et al., 2019
	Dean et al., 2020

CSC-enriched spheroid models	Lim et al., 2011
	Goričan et al., 2020
	Melissaridou et al., 2019

Patient-derived explant models	Robbins et al., 1994
*-in vitro-*	Au et al., 1993
*-in vivo-*	Pathak et al., 2007
	Gerlach et al., 2014
	Suzuki et al., 2015
	Freudlsperger et al., 2015
	Donnadieu et al., 2016
	Affolter et al., 2016
	Peria et al., 2016
	Hickman et al., 2014
	Dohmen et al., 2018
	Engelmann et al., 2020
	Peng et al., 2013
	Li et al., 2016
	Klinghammer et al., 2015
	Facompre et al., 2017
	Morton et al., 2016
	Stecklum et al., 2020

Tumor on chip	Bhatia and Ingber, 2014
	Skardal et al., 2017
	Aleman and Skardal, 2019
	Carr et al., 2014
	Kennedy et al., 2019
	Bower et al., 2017
	Sylvester et al., 2013

Bioprinting models	Almela et al., 2018

Mathematical modeling for precision medicine	Linxweiler et al., 2016
	Panikkanvalappil et al., 2019
	Emerick et al., 2013
	Enderling et al., 2019
	Caudell et al., 2017
	Scott et al., 2017

*CSC, cancer stem cells; HNSCC, Head and neck squamous cell carcinoma.*

### Three-Dimensional Culture Models/Spheroids

Spheroids have widely gained popularity because they reflect the tumor architecture more precisely compared to 2D systems or monolayer cultures. A tumor grows in a 3D spatial array therefore cells incorporated in these tumor formations are not evenly exposed to nutrients and oxygen as well as to cellular stress factors. The TME varies between the single regions of a 3D tumor construct. It is clear that in a 2D assay all tumor cells are exposed to oxygen and nutrients in an equal manner. Thus, in this system the factual situation in cancer is misrepresented and is only suitable to assess a minor fraction of HNSCC molecular biology aspects. The impact of cell-cell contacts on therapeutic response is not depicted in monolayer cultures; the informative value of *in vitro* treatment efficacy studies is limited (Figure 1).

When analyzing treatment sensitivity, considerable differences were observed between 2D and 3D HNSCC *in vitro* cultures in response to irradiation (Storch et al., 2010) and EGFR inhibitors or cisplatin (Melissaridou et al., 2019). Interestingly, Melissaridou et al. reported an increased resistance to cisplatin treatment, which might be due to increased expression of CSC-associated proteins Nanog and Sox1 observed in tumor spheroids. They conclude that cells cultured in 3D take on the CSC-like phenotype and that lower sensitivity of cells cultured under 3D conditions may be attributable to this increase expression of genes associated with epithelial-mesenchymal transition (EMT) and stemness (Melissaridou et al., 2019).

Multicellular tumor spheroids (Franko and Sutherland, 1979; Köpf-Maier and Zimmermann, 1991) have been established for various entities including HNC already decades ago. Spheroids have an outer layer of proliferating cells and an inside layer of mainly quiescent cells with necrotic areas in the center and can be further divided into different subpopulations (Carlsson and Nederman, 1989). In 2012, it was demonstrated that there is great similarity of EGFR signaling and radiation response between 3D SCC cultures and HNSCC xenografts compared to cell monolayers (Eke et al., 2013). The authors describe a greater physiological relevance of 3D growth conditions regarding cell morphology, gene and protein expression patterns, protein-protein interactions, and intracellular signaling as well as response to radio/-chemotherapy (Kenny, 2007; Eke et al., 2009; Storch et al., 2010).

These 3D models have been shown to phenocopy the original tumor in a way that *in vitro* drug responses of the model can be traced back to genetic alterations of the primary (Drost and Clevers, 2018). Hagemann et al. (2017, 2018) propose a single spheroid-based *in vitro* assay, which is described as a testing tool for individual therapy susceptibility to current standard and new targeted regimens. Spheroids were generated from single cell suspensions, first from different cell lines including the proprietary PiCa cell line, later from primary human cancer cells derived from fresh tumor biopsies. Spheroids were exposed and incubated with cisplatin or 5-FU, which led to a significant reduction in growth speed over time compared to untreated controls. Spheroids were also irradiated with a dose of 2 Gy significantly reducing the dynamic of cellular growth. They claim their spheroid model to be a useful tool to unveil drug effectiveness and complex drug resistance mechanisms. Factors impacting on radiation response such as DNA repair, apoptosis, oxygen supply and cellular contacts in spheroid cell culture are considered as comparable to *in vivo* tumors (Dubessy et al., 2000; Weiswald et al., 2015). Although 3D cultures such as spheroids have advantages compared to monolayer techniques, the system has inherent limitations. Although spheroids are established for being exposed to different conventional and novel therapies with cell viability and spheroid size as read-outs these experiments and moreover the correlation with clinical data and prognosis are still pending. The model is most likely a straight forward and cost-efficient assay but it is unclear how predictive it is in terms of correlation with the later response to therapy and with clinical outcome (Hagemann et al., 2017, 2018).

Extensive analyses on and detailed culture conditions for oral mucosal organoids were recently published by Driehuis et al. (2019). They managed to keep a panel of 26 organoids deriving from HNSCC and corresponding normal epithelium in culture for 10–14 days. The organoids showed rapid growth and culture exceeded 65% efficiency. Productive infection of the organoids with herpes simplex virus (HSV) and HPV could be demonstrated which has so far only been described for immortalized cell lines, or primary cells with short cultivation periods. The susceptibility to common drugs such as cetuximab, the PI3K inhibitor alpelisib, and the BRAF inhibitor vemurafenib was also assessed. In summary, the tumor cell phenotype, the tumorigenic potential, and the response to therapy were assessed by the use of the 3D model. Altogether clinical response data of seven HNSCC patients were analyzed. In one case, a correlation between radiation sensitivity of the HNSCC organoids and the clinical response of the corresponding HNSCC patient could be observed. However, the authors define the aim of the study as of establishing optimal conditions as an experimental basis for future prospective investigations of larger tumor cohorts, not to determine the predictive potential of HNSCC-derived organoids in therapeutic guidance.

Cancer stem cell-enriched spheroids incorporate CSC or cells with stem cell traits. The expression of CD44 detected by cell sorting or self-renewing features is used for enriching these CSC. Weiswald et al. (2015) describe tumorospheres as a model of CSC expansion established in a serum-free medium with stem cell media growing under low-adherent conditions. In this model it has to be ensured that the self-renewal capacity of the CSC is depicted. Goričan et al. describe a new HNC stem cell-enriched spheroid model (SCESM) suitable for high-throughput screens of anti-CSC compounds. Advantages are that the model is faster than the traditional culture of free-floating spheres and enables higher CSC enrichment than the multicellular spheroid model (Goričan et al., 2020). Melissandrou et al. found that under 3D culturing conditions cells adapt to a CSC-like phenotype. They observed that results from 3D HNSCC culture differed significantly from monolayer data (Melissaridou et al., 2019). HNSCC-driven sphere-forming cells (referred to as squamospheres) that possess the general properties of CSC, such as self-renewal, stem cell marker expression, aberrant differentiation, tumor-initiating potential, and chemoresistance with increased side population have been reported by Lim et al. (2011) who interpret their model as an aid for new insights into CSC biology in HNSCC.

However, the usability of CSC-enriched spheroids is limited by the fact that the original tumor is not histologically preserved. Furthermore, CSC frequently develop a central necrosis (Demers et al., 2020). Putative CSC or tumor cells exhibiting stem-cell like features for the use of 3D cultures have been obtained from tumors. As CSC cultivation is determined by certain in-depth conditions which these experiments do not necessarily adapt why results from CSC-enriched spheroid cultures should be accepted with reservation (Ishiguro et al., 2017).

The evaluation of self-renewal can be hampered by cell density as the clonal growth conditions are impaired. The comparability of different studies on CSC-enriched spheroids is limited because of the variation of experimental parameters such as sphere size. However, it is anticipated that after refinement of protocols and SOPs tumor-derived cultures will be a useful tool for the identification of CSC-inhibiting molecules. Especially in the light of liquid biopsy approaches, spheroid cultivation of CSC obtained from circulating blood may allow to analyze the original tumor in a non-invasive manner (Ishiguro et al., 2017) (Figure 1).

### Head and Neck Cancer Models Preserving the TME – 3D Co-culture Tumor Models

There are many benefits from current models such as organoids but although these models have been shown to phenocopy the original tumor in terms of drug susceptibility they lack the preservation of tumor-stroma interactions which are known to be essential for carcinogenesis and even resistance development (Storch et al., 2010; Valkenburg et al., 2018). Sensitivity testing of novel immunotherapeutic approaches is impaired as they require an intact immune system to be effective. Results from co-culture studies (Sawant et al., 2016) give clear evidence that the TME exerts tumor-promoting effects (Curry et al., 2014) which is a strong argument for the establishment and implementation of more advanced 3D tumor models (Tinhofer et al., 2020). Cancer-associated fibroblasts (CAFs), one of the major components in TME, play a critical role in tumorigenesis. On the one hand, there is an active interaction of CAFs with tumor cells, with CAFs promoting survival and invasion properties of cancer (Xu et al., 2014; Young et al., 2018; Rodrigues et al., 2020). CAFs create a tumor-supportive environment but do not only affect tumor cells but also immune cells through direct and indirect mechanisms (Takahashi et al., 2016; Huang et al., 2019). However, more interrelations need to be extensively studied to better understand the role of CAFs in HNC. For instance, CAFs might play a different role in HPV-driven HNSCC compared to non-HPV driven HNSCC, but these aspects are still unclear. As tumor-associated stromal cells exert either positive or negative effects on tumor growth and propagation, the absence of the stromal components in multicellular tumor spheroid models limits the utility of such models in cancer research (Xu et al., 2014). 3D cell culture models incorporating the tumor in its entirety are therefore clearly needed to illustrate dynamic and reciprocal interactions between the solid tumor and the TME, including immune cells, stromal cells, stromal ECM components, and growth factors (Figure 1).

Young et al. propose the TRACER model (Tissue Role for the Analysis of Cellular Environment and Response) as a co-culture protocol simulating interactions between CAF-TME in head and neck tumors. The original basic monoculture TRACER system is a stacked paper tumor model in which cells in a hydrogel matrix are infiltrated into a cellulose scaffold that is rolled onto an aluminum core to reflect a 3D construct. The authors have refined the former monoculture by incorporating both head and neck primary patient CAFs and head and neck tumor cell populations in order to assess tumor-CAF interrelations. The longest culture time that was assessed for the co-culture TRACER configuration is 48 h as it was assumed that cancer cells would outgrow CAFs in a direct co-culture construct under extended cultivation periods. The authors who were using an immortalized HNSCC cell line (FaDu) speculate that primary cancer cells would be essential to avoid outgrowth and also to reflect heterogeneity of the patient population. The model is considered to be feasible for reflection of migration patterns of tumor cells through the CAFs environment and for mimicking CAFs‘ influence on tumor cells. However, the authors consider the fact that the system is not entirely suitable to address questions that require real-time imaging. Furthermore, while some features of the TME can be recreated, relevant structures such as a basement membrane and other important cell types are not represented in the platform (Young et al., 2018). The cultivation period is rather short with only 48 h because of the anticipated outgrowth of tumor cells that might cause a disproportionate ratio of the two cell populations. The authors envision that incorporation of primary cancer cells in the system will be necessary for longer cultivation periods and also to reflect heterogeneity of patient tumors. The model was supposed to be valuable for exploring the impact of hypoxia which is known to alter the behavior of stroma and cancer cells toward progression of disease (Brown and Wilson, 2004; Vaupel et al., 2004; Vaupel and Mayer, 2007). Conventional 3D co-culture models are mostly unsuitable to assess questions where spatial organization of different cellular components within the tumor is relevant. The TRACER platform, however, is assumed to be useful for patterning of distinct cellular compartments and cell separation for phenotypic assessment (Young et al., 2018).

Dean et al. expand the TRACER model by co-culturing HNSCC cells with CAFs and found that tumor cell invasion into an acellular collagen layer was enhanced in this co-culture setting. The observation was found to be dependent on the distribution of ECM density within the culture. The platform allowed to mimic density variations of tumors *in vivo* and to reflect the distribution of CAFs within the tumor at different disease stages (Dean et al., 2020).

Hoffmann et al. demonstrated that an anti-EGFR monoclonal antibody induced leukocyte infiltration into EGFR-overexpressing tumor spheroids due to an upregulation of chemokine expression mediated by anti-EGFR signal transduction. The spheroids enabled them to identify molecular mechanisms responsible for anti-EGFR monoclonal antibody-mediated infiltration of immune cells into tumors (Hoffmann et al., 2009).

### Patient-Derived Explant Models

Patient-derived explants may be beneficial as those systems retain the original TME including the ECM, and stromal, immune, and vascular cells, thereby reflecting tumor stroma interactions. Powley et al. (2020) claim PDEs to be a potent model of choice for cancer drug and biomarker discovery programs taking tumor heterogeneity into account. However, the application of PDEs has not been implemented in the daily clinical routine yet. We recently described the maintenance of HNSCC tumor explants on a dermal equivalent (DE) for up to 21 days, which is the longest culture duration demonstrated so far (Demers et al., 2020; Engelmann et al., 2020). The DE containing human fibroblasts is used as a scaffold for cultivating vital HNSCC samples. One of the main findings was that the model allowed to classify the tumor samples into different invasion patterns, namely invasive, expansive, and silent. The invasive type shows an infiltrative scattering of detached tumor cell clusters into the scaffold. Expansively growing tumor samples migrate horizontally on top of the DE, while silent type tumors grow without migration or invasion. CAFs and leucocytes could be consistently detected for up to 21 days representing the modeling of an intact TME. The new system allows to mimic fractionated irradiation showing heterogeneous responses within the cohort, measured by expression levels of apoptosis marker cleaved caspase-3 as a read-out. Interestingly, one patient suffering from an early recurrence 17 months after first diagnosis and treatment, with the corresponding 3D-OTC sample displaying invasive growth which might reflect a more aggressive tumor biology. Although it was feasible to maintain HPV-driven HNSCC in culture for up to 21 days the interpretation of results was more challenging, with varying p16INK4a expression levels over time (Engelmann et al., 2020).

Dohmen et al. present a sponge-gel-supported histoculture model, which has been developed for prediction of individual responses to therapy. Tumor fragments were kept in culture for 7 days The proportion of tumor cells could be quantified, tumor viability, proliferation, EGFR expression levels and present immune cells were scored The authors emphasize the sustainability of the microenvironment they found with immune cells still present on day 7 as one of the major advantages of their model. Although it was unclear whether they were still functionally active or viable, based on the morphology the authors believe the immune cells to keep their viability up to day 7. The authors hope that the histoculture model, which comes closer to reality than cell lines or even organoids due the preservation of the heterogeneity and TME may allow for personalized treatment stratification and testing for new treatment strategies in the future (Dohmen et al., 2018).

In the past few years the *ex vivo* tissue culture model was established. The model allowed to prove the increased activation of the ERK/MAPK and PI3K-AKT signaling cascades by irradiation and its modulation by pharmacological inhibition (Freudlsperger et al., 2015; Affolter et al., 2016). A heterogeneous induction of ERK and AKT phosphorylation was found in the tumor tissue cultures suggesting a contextual regulation mode. Due to the small number of cases, correlation with clinical parameters was possible only to a limited extent. However, patients with low basal ERK phosphorylation and postradiogenic induction suffered from a relapse during follow up suggesting that this constellation might be an indicator for an unfavorable outcome (Affolter et al., 2016).

Peria et al. describe the cultivation of explant HNSCC tissue cultures over 48 h in order to immunohistochemically evaluate the expression of Ki-67, AE1, AE3, p40, and CK-5/6 after administration of cetuximab or sorafenib, respectively. During this time frame tumor architecture and cell viability were sustained. Afterward, the tissue increasingly developed necrosis and the effect of treatment with cetuximab based on few remaining proliferating cells could no longer reliably be analyzed. Clinical patient data were not matched with the results from the explant cultures. This is planned for follow-up studies in bigger cohorts (Peria et al., 2016).

Despite encouraging results described by us and others PDE cultures are limited by the following factors as summarized by Powley et al. (2020): PDEs can only be cultured after extraction of sufficient spare tumor material either by surgical resection of the tumor or tumor biopsies. Fresh tumor tissue for experimental procedures needs to be obtained before the surgical specimen is formalin-fixed for pathologic routine diagnostics. That means, the excision of tumor material for experimental procedures must by no means affect clinical diagnostics. The access and preparation of tumor tissue is dependent on the collaboration with head and neck surgeons, OR staff, and pathologists. Last, if the tumor tissue is not intact anymore, experimental data can be impaired. Therefore, Powley et al. list PDEs as a poor model for invasion and metastases, however, we have recently featured a 3D-organotypic co-culture model for HNSCC as a tool to study different invasion patterns possibly correlated with clinical characteristics and outcome We did not detect any disintegration of the tumor architecture during this period (Engelmann et al., 2020). Peria et al. referred to the currently required relatively large amount of fresh tumor tissue as a disadvantage of short-term assays. In small HNSCC there is a risk of rendering the margins of the specimens unusable for the pathologist when preparing vital tissue cultures. The authors aim to adapt their technique to the use of needle biopsy material to generate higher case numbers (Peria et al., 2016).

Hickman et al. (2014) criticize that a standardization of the manufacturing process could be hampered by the variability of individual steps such as ischemia times of the tissue and time window to start the cutting procedure. Mechanical stress factors in the course of sample processing, such as changes of temperature, the oxygen content, and availability of nutrients, not only determine the tissue quality at the starting point of the cultivation, but are likely to influence both the sensitivity of the tumor cells *ex vivo* on therapeutics as well as their suitability for reliable biochemical analyzes. So far, these issues appear to pare down most of the tissue cultures approaches to short-term experiments (Figure 1).

Possible solutions are, on the one hand, combinations of strategies, to achieve longer cultivation times, such as scaffolding the vital tumor explant by distinct matrices as a co-culture model (Engelmann et al., 2020). On the other hand, the comparison of preparation and culture conditions in multicenter validation studies for simplification of processes is a prerequisite to find a broad application in patient stratification for novel, yet to be defined therapy regimens. Once there is a standard protocol, expression analysis of central markers for pathogenesis and for treatment response in HNSCC should be evaluated to test their sensitivity to combination schemes consisting of new and conventional treatments.

Patient-derived xenograft (PDX) models are being applied for various tumor entities including HNSCC (Fichtner et al., 2008; Daniel et al., 2009; Peng et al., 2013; Lin et al., 2014; Li et al., 2016). They are generated by implantation of tumor cells derived from fresh patient tumor tissue subcutaneously, orthotopically, or under the kidney capsule of immunodeficient mice (e.g., NODSCID, NSG mice) (Choi et al., 2014). Conventional xenograft models with established tumor cell lines often show little histological similarity to the original tumor, due to *in vitro* co-cultivation and associated changes in the genetic and epigenetic profiles (Daniel et al., 2009). It is feasible to analyze different compounds and their combinations through passaging and transplantation of the tumor in several mice. It has been observed in several studies that PDX models do predict clinical trial drug responses (Bertotti et al., 2011; Gao et al., 2015; Townsend et al., 2016; Karamboulas et al., 2018).

At least initially, PDX models represent complex biological and molecular interactions between tumor cells and their microenvironment (Garber, 2009; Tentler et al., 2012). Furthermore, phenotypic and molecular characteristics of the original tumor tissue such as chromosomal copy number variants, single nucleotide polymorphisms and gene expression profiles, are mapped (Choi et al., 2014). Cassidy et al. criticize that stromal influences on therapy response as well as immunologic drivers remain underrepresented in the PDX model. In the course of passaging, the PDX parts of the human stroma are replaced by murine equivalents, and it is unclear how precisely mouse fibroblasts mimic their human counterparts (Cassidy et al., 2015). Another main challenge of PDX models is the lack of a functional human immune system to analyze tumor-TME-interactions, especially in terms of immuno-oncological questions. High costs and high personnel expenditure also hinder the widespread use of the model system (Choi et al., 2014). Moreover, the engraftment of HPV-driven HNSCC compared to non-HPV driven HNSCC appears to be complicated as reported in various studies (Klinghammer et al., 2015; Facompre et al., 2017).

A possible solution for the lack of a functional immune system in the PDX model is the humanized mouse model (XactMice model) for HNC (Morton et al., 2016). In this system, human hematopoietic stem and progenitor cells (HSPCs) reconstitute the bone marrow from NSG (NOD/SCID/IL2rg-/-) mice that has previously been suppressed by irradiation. Consecutively, patient-derived tumor tissue is transplanted into the mice. Human HSPCs form immune cells that invade into the xenograft and recreate its natural microenvironment. In consequence, the expression patterns of epithelial, stromal, and immunological genes in XactMice tumors match the patient’s tumor to a greater extent than tumors from non-humanized mice. Likewise, iPDX (immune-patient-derived xenograft) models offer the possibility of testing the effects of CPI on the human immune system. In this variation of the conventional PDX platform the experiments are performed during the first passages, before the replacement of human by murine stroma. By systemic administration of monoclonal antibodies to the animals, human TILs in the TME can be targeted. So while it is advantageous to explore the species-specific interaction among human tumor and immune cells, broad clinical applicability is limited by the fact that iPDX only start growing after 1–2 months and the number of mice that can be xenografted per sample is low (Sanmamed et al., 2016). Approaches for HNSCC immune-PDX models have recently been proposed (Stecklum et al., 2020). However, there is no resounding draft for the application of PDX models in the clinical routine so far.

## Real-Time Live Imaging in Precision Medicine

Molecular imaging is a real-time and non-invasive approach for visualization of expression and activity of relevant targets as well as various biological processes, namely hypoxia, angiogenesis, and apoptosis. The ambitious aim is to streamline progress into novel drug development approaches by discovering physiologic or molecular alterations prefiguring cancer at an early stage with better prognosis, and depicting response to cancer therapy in a real-time setting (McDermott and Kilcoyne, 2016). New optical technologies such as confocal laser endomicroscopy (CLE) help to overcome invasive and time-consuming surgical biopsies. CLE enables microscopic visualization of lesions in real-time (optical biopsy) for different cancers, including HNSCC (Linxweiler et al., 2016). From their study on 10 HNSCC tissue samples Shinohara et al. describe a clear difference between cancer and normal mucosa in the uniformity of nuclear size and shape measured by real-time *in vivo* imaging using CLE. The technique could potentially be usable for the distinction of cancerous from non-cancerous tissue without invasive biopsies. Although the authors point out to several limitations, such as the examiner’s bias and their improvable double staining method, it is likely that CLE will gain relevance for in real-time classification of regions in the head and neck (Shinohara et al., 2020). Panikkanvalappil et al. describe plasmonically enhanced Raman spectroscopy (PERS) in the real-time monitoring of endogenously generated CO and assessing the dynamics of hemeoxygenase-1 (HO-1) in live HNC cells. Their findings could produce useful insights into the signaling action of CO and HO-1 in tumor progression (Panikkanvalappil et al., 2019). Lee et al. established intravital imaging models to enable real-time observation of cancer cells of the bone marrow environment. In an approach to identify the biologic processes of cancer cells in a real-time manner the distribution and phenotype of cancer cells in bone marrow of a live mouse model could be assessed by two-photon microscopy. The study provided new data about *in vivo* cancer cell biology in bone marrow. In particular, the group identified dormancy and reactivation of cancer cells. Interestingly, after injection of the chemotherapeutic agent gemcitabine, cancer cells appeared to be less affected than normal cells in the bone marrow. However, the technique has certain disadvantages, as the scope to perivascular niche for the cancer cells in the bone marrow environment is limited and wound healing processes as induced by bone marrow transplantation, might impair the results. The model is promising but has to be experimentally validated (Lee et al., 2018). To date, for HNSCC there is no comparable real-time live imaging model.

## Sensitivity Testing in Histoculture Models

Already 25 years ago approaches were taken to assess the correlation between drug sensitivity of sponge-gel-supported histocultures and their corresponding original HNC specimens to cisplatin (Robbins et al., 1991, 1994; Au et al., 1993). The *in vitro*-like maintenance of three-dimensional tissue architecture of the tumors in histoculture was supposed to confer clinical predictivity of drug response of the model (Robbins et al., 1994). The Histoculture Drug Response Assays (HDRA) is a three dimensional native state histoculture assay that simulates the structure of tumor in the body and is used to assay the chemo-responsiveness of the tumor. One major limitation of these HDRA is their comparatively short cultivation period (Robbins et al., 1991, 1994). HNSCC histocultures have been tested for response to various chemotherapeutics, such as cisplatin, 5-FU, and mitomycin C treatment. While there were no apparent differences for cisplatin and mitomycin C, the nodal metastatic tumors seemed to be less sensitive to 5-FU than the primaries (Au et al., 1993). A more recent study by Gerlach et al. tested the response of vital HNSCC slides on membrane culture inserts to cetuximab, cisplatin, and docetaxel and observed cell loss and also an increase of apoptosis reflected by an increase of cleaved caspase 3 after treatment with docetaxel but not with other drugs. The authors concluded that their assay can be used to better understand the mechanisms of tumor resistance by harvesting surviving tumor cells after treatment. However, they did not correlate susceptibility to treatments of the cultured tumor samples to the respective patient’s response and clinical outcome yet (Gerlach et al., 2014).

Indeed there was one study in which patients received the same treatment that was tested *in vitro*, by the use of a HDRA (Pathak et al., 2007). Inhibition rates quantified by proliferation assays were taken as a read-out. There was a significant correlation between the *in vitro* chemosensitivity and the clinical response to different chemotherapeutic regimens.

Although these data appear promising, there are various reasons why the HDRA is not taken into routine clinical practice such as the requirement of an in-hospital laboratory to ensure the quick processing of fresh biopsies, large effort of time and personnel and last the lack of sensitivity and specificity when correlating *in vivo* tumor response to *in vitro* HDRA chemosensitivity (Dohmen et al., 2018).

In terms of survival prediction, Suzuki et al. investigated the correlation of overall survival represented by 18F-FDG-uptake with *in vitro* sensitivity data. 18F-FDG-uptake of the primary tumor in PET/CTs, which is considered as a marker of poor OS, and *in vitro* chemosensitivity of cisplatin using HDRA in HNC were correlated. In samples with a high inhibition index of cisplatin in the patient cohort a prolonged OS was significantly associated with a high inhibition index (>50) and a high SUV max (standardized uptake value) validated by PET (Suzuki et al., 2015).

Recently, Driehuis et al. reported a BRAF-mutant organoid line derived from a BRAF-mutant HNSCC to show increased responsiveness to the BRAF inhibitor vemurafenib. However, no correlations between EGFR expression of the organoids and cetuximab response were found when cetuximab was applied or between sensitivity toward PI3K inhibitor alpelisib and organoids harboring a PIK3CA mutation (Driehuis et al., 2019). Donnadieu et al. report the short-term cultivation of HNSCC tumor slices for 48 h. Cultures were exposed to a panel of targeted therapies that are directed against selected oncogenic transduction pathways, including EGFR, B-RAF, KIT, HGFR, FGFR, and mTOR. The compounds were in phase II/III trials for the treatment of solid malignancies at the time the study was conducted. The authors observed varying responses to the different treatments in the individual patient samples. Proliferation was impaired by multi-kinase inhibitor sorafenib in 5/14 of the individual patient samples while, interestingly, cetuximab as the only drug approved for HNSCC treatment was only effective in 2/14 tumors. At least one of the eight drugs tested caused a more than 50% inhibition of proliferation in 10/14 tumors (Donnadieu et al., 2016).

## Personalized Therapeutic Approaches for HNC Based on 3D and Stem Cell Models and Implementation Into Clinical Routine

Why has none of the novel and auspicious efforts made it into the clinical routine so far? Blom et al. discuss the lack of evidence in the form of prospective correlations as a reason. No randomized controlled trials have been performed to compare assay-led treatment with standard or physician’s choice of therapy regarding prolongation of survival (Blom et al., 2017).

Precision medicine initiatives applying small molecule inhibitors (SMIs) and monoclonal antibodies may reveal significant potential for the management of not only HNSCC but also malignancies of the salivary glands. Still, targeted therapy studies have not shown any sweeping success in HNSCC so far (Machiels et al., 2010; Schmitz et al., 2012; Vokes et al., 2015; Kochanny et al., 2020; Seiwert et al., 2020). Preclinical tumor models provide the opportunity to test targeted therapies on patient tissues. However, as mAbs and SMIs except cetuximab are not implemented in the clinical routine treatment of HNSCC yet, the correlation of experimental results with clinical data and outcome is practically impossible. This may be one explanation why the predictive potential of 3D cancer models has not been validated in large patient populations so far.

Different diagnostic tests are routinely used to predict the activity or resistance of some targeted therapies. Molecular profiling of the individual tumors is performed in the frame of biomarker-driven studies but to unveil mechanisms of resistance development is not considered in these studies (Von Hoff et al., 2010; Tsimberidou et al., 2012, 2017; Massard et al., 2017; Harris et al., 2018). Some trials select patients upfront on the basis of genetic alterations which are generally rare, for instance, when treating patients with FGFR-positive recurrent HNC with Infigratinib (BGJ398) (NCT02706691) or screening patients for HRAS mutations to allocate them to treatment with Tipifarnib (NCT03719690). Although the medication arms in these trails seem to yield promising results, the trials bear the risk of a large proportion of screen failures as they assign patients to a very specific therapy for rather small subpopulations harboring those rare mutations.

The EORTC-1559-HNCG (NCT03088059) is an umbrella biomarker-driven study dedicated to recurrent and/or metastatic HNSCC patients for second-line treatments and beyond after platinum-based therapy (Galot et al., 2018). First, NGS and IHC, respectively are carried out on actual biopsy material in order to depict oncogenes and tumor suppressor genes relevant for HNSCC. These findings are the basis for the inclusion of patients to one of the treatment arms or in case of non-eligibility to an immunotherapy cohort (Galot et al., 2018).

So-called window-of-opportunity clinical trials aim to identify biomarkers in tumor samples that have been collected after application of a certain drug for a short period of time and before definitive therapy, while efficacy is not necessarily expected. This period is called a window-of-opportunity situation. It is an advantage that post-treatment samples can be easily collected from the surgically removed specimen. However, window-of-opportunity trials have been rare, especially in HNSCC (Marous et al., 2015; Gougis et al., 2019).

More umbrella and basket trials are currently under way for HNSCC to investigate targeted compounds in monotherapy (Galot et al., 2018; Gougis et al., 2019). However, tumor heterogeneity is one of the most notable features of HNSCC is not taken into account. Hence, translational research focusing on the analysis of liquid biopsies and tumor tissue samples and building bridges between bench and bedside is crucial for identifying resistance mechanisms and subsequently new combinatorial regimens that are potent to overcome them.

In this regard, it should be considered that in HNSCC DNA-guided precision medicine is not the only aspect to be taken into account. The genotype alone may not reliably predict drug responses suggesting that the tissue in which the cancer mutation occurs can be a major factor in determining response to therapy (Cohen and Settleman, 2014; Voest and Bernards, 2016).

As discussed before, sensitivity testing in histoculture models is not novel in HNSCC research. In 2001, Singh et al. already claimed the HDRA to be a strong predictor of survival. There was a significant association between HDRA assessment of chemoresponse and clinical outcome in 41 HNSCC samples treated with cisplatin and 5-fluoruracil. Afterward, cell survival fractions were determined by proliferation assays. A tumor was defined sensitive when the inhibition rate was greater than 30%. Patients whose tumors were sensitive toward one or both of the chemotherapeutic agents had a more favorable outcome in terms of cause-specific survival at 2 year follow up (Singh et al., 2002). In a more recent study, Jamal et al. demonstrate results from a retrospective analysis of 22 HNSCC specimens that underwent testing with the ChemoFx assay (Precision Therapeutics, Inc.). The assay is supposed to determine the response to chemotherapy thereby serving as a prediction tool. Tumor cells were isolated from solid tissue samples and cultured, then exposed to increasing dosages of conventional chemotherapeutic agents. Response was measured in quantifying cell counts and classified as “responsive,” “intermediate response,” or “non-responsive.” Data from 11/21 patients were eventually interpreted. Nine of those showed a predictable chemoresponse assay (81.8% predictability of effective treatment). All 6 patients who had a predictable poor response failed their chemotherapeutic regimen within 6 months and succumbed to their illness after 3 years of follow up, except one patient with an intermediate response in the assay. The authors interpreted the test to having the potential as a useful adjunct for the selection of therapeutic schemes while acknowledging its limitations (Jamal et al., 2017). Although the data are quite promising, this model is certainly limited by the absence of TME cells impacting on treatment response. The patients which were treated with combined surgery and radiochemotherapy (RCT), while cultures were merely treated with cisplatin although it is known that irradiation and cisplatin show synergistic effects with platinum-based compounds functioning as a radiosensitizer (Negi et al., 2016). RT was not modeled in cultures though.

So far, none of the platforms discussed here has made it into the daily routine of HNSCC treatment. In this regard, Blom et al. (2017) point to another challenge of preclinical tumor platforms. They claim that (chemo) sensitivity assays might be a better predictor for therapy resistance than for sensitivity. Complex mechanisms of resistance development *in vivo* may go beyond those at the cellular levels and cannot be modeled *ex vivo*. Consequently, sensitivity is expected to be higher than specificity in these assays.

To achieve an acceptable correlation with clinical treatment schemes, various combinations regimens administered to patients must be diligently imitated *ex vivo* to ensure data comparability. However, validation of existing tumor models can only be obtained in large prospective trials which are still outstanding to date.

## Cancer Stem Cells in Therapy of HNSCC

Cancer stem cell share properties with somatic stem cells such as the ability to self-renew and differentiate. Furthermore, CSC are thought to be non-responsive to antineoplastic treatments such as chemo- or RT and are therefore clinically decisive. As with somatic stem cells, CSC are thought to reside in a specialized supportive microenvironment called the stem cell niche. Possible strategies in HNSCC-therapy could be affecting functions of the stem cell niche or target CSC themselves. Further factors contributing to CSC therapeutic resistance include the activation of signaling pathways that provoke self-renewal; the presence of multiple drug resistance membrane transporters (e.g., ATP-binding cassette drug transporters) and an immense capacity for DNA repair. All of these targets hold the potential to serve as a therapeutic strategy for different cancer entities including HNSCC.

### Targeting the CSC Niche

The interaction between SDF-1 and its receptor CXCR4 could play an important role in the environment of the tumor stem cell niche. SDF-1 is a multifunctional cytokine that is secreted by a wide variety of tissues, including endothelial and stromal cells (Chen and Wang, 2019) 282 nucleotides code for a polypeptide of 93 amino acids. SDF-1 occurs in two isoforms through alternative splicing, SDF-1 alpha (amino acids 24–88) and SDF-1 beta (amino acids 24–93) (De La Luz Sierra et al., 2004; Faber et al., 2013a). SDF-1 alpha is so far the only proven chemokine which is capable to induce migration in hematopoietic progenitor cells (Möhle et al., 1998). Accordingly, it is considered one of the key regulators of hematopoietic progenitor cells in the cell traffic between peripheral blood circulation and bone marrow. We and others previously indicated that SDF-1 alpha induces polarization and directed migration of hematopoietic progenitors and leukemic cells (Möhle et al., 1998; Faber et al., 2007), two requirements for metastasis. The 7-transmembrane receptor CXCR4 was identified as the receptor of SDF-1 alpha. Interactions between SDF-1 alpha and CXCR4 already play a role in embryonic development, especially in the hematopoietic, vascular, and cardiac systems. The signal transduction pathways triggered by the binding of SDF-1 alpha to CXCR4 are not yet fully understood. Mechanisms that are involved here include Gi protein-supported activation of PI3K and the phospholipase C cascade (Petit et al., 2005; Goichberg et al., 2006).

The function of SDF-1 alpha, which is bound to CXCR4, may be mimicked or blocked by small peptide agonists or antagonists (Faber et al., 2007). Affecting signal transduction pathways activated by the SDF-1-CXCR4 axis by peptide agonists (or antagonists) includes a high potential for therapeutic interventions. There is increasing evidence that cell migration along with adhesion to the cellular stem cell niche is important in HNSCC CSCs. To unveil these mechanisms could potentially facilitate novel therapeutic options – e.g., through the use of peptide agonists and antagonists (Faber et al., 2007) for interference with the tumor stem cell niche and the subsequent inhibition or blockade of further tumor invasion and metastasis.

Our own preliminary work has already demonstrated the existence and functionality of this axis in the tumor stem cell niche of HNSCC (Faber et al., 2013b).

### Targeting CSC Signaling Pathways

Erroneous signaling pathways can result in formation of CSC populations leading to tumor recurrence or metastasis. Disturbance of physiological pathways that are involved in the regulation of normal stem-cell-renewal, proliferation, and differentiation can promote tumorigenesis. When aberrantly activated, the Hedgehog (HH) signaling pathway, that is essential for stem cell maintenance, has been implicated in the tumorigenesis of various malignancies. Activating mutations in the HH pathway cause a subset of skin (basal cell carcinoma) and brain (medulloblastoma) tumors. Furthermore, the growth of many tumors is supported by HH pathway activity in stromal cells (Ng and Curran, 2011). HH inhibitors, which can be naturally occurring or synthetic, block both intrinsic signaling in cancer cells as well as extrinsic signaling to stromal cells to reduce tumor growth (Yauch et al., 2008). In HNSCC, Gan et al. showed that radiation-induced therapeutic inhibition by increased glioma-associated oncogene GLI1 expression could be partly reversed by HH pathway blockade with cyclopamine which resulted in radiosensitivity of the tumor. They demonstrated that GLI1, that is upregulated at the tumor-stroma intersection in HNSCC, is increased by RT, where it contributes to stroma-mediated resistance, and that HH inhibition offers a rational strategy to reverse this process and to sensitize HNSCC to irradiation (Gan et al., 2014).

The Notch signaling pathway is important for stem cell proliferation, differentiation, and apoptosis (Wang et al., 2008). It is activated through ligand-receptor-interactions (Notch receptors Notch 1–4 and Notch ligands Delta1, 3, 4 and Jagged1, 2) and known to play a role in a variety of malignancies, e.g., breast cancer and glioma (Krishna et al., 2019; Parmigiani et al., 2020). Inhibition of the Notch pathway with specific antibodies directed against Notch-ligands or –receptors has been shown to reduce breast CSC populations and to improve efficacy of chemotherapy in PDXs (Hoey et al., 2009; Qiu et al., 2013). Zhao et al. found elevated levels of Notch1/Hes1 in human HNSCC, especially in tissue post chemotherapy and lymph node metastases. The Notch1-inhibitor DAPT (GSI-IX) significantly reduced CSC populations and tumor-self-renewal ability *in vitro* and *in vivo*. The combined strategy of Notch1-blockade and chemotherapy synergistically attenuated chemotherapy-enriched CSC populations, promising therapeutic potential in future clinical trials (Zhao et al., 2016).

In HNSCC, the Wnt signaling pathways could also comprise therapeutic starting points. The Wnt/beta-catenin pathway is well characterized and there is increasing evidence indicating its role in oncogenesis and tumor development (Takahashi-Yanaga and Kahn, 2010). Wnt pathway activation maintains CSC phenotype and promotes tumor progression e.g., Wnt activation increases CSC characteristics like sphere formation and invasiveness. Accordingly, Wnt inhibitors significantly reduce growth of HNSCC PDXs and suppress Wnt activation at the tumor epithelial-stromal boundary (Le et al., 2019). These studies suggest targeting Wnt signaling in the TME might offer a promising therapeutic approach.

The transforming growth factor-beta (TGF-β) superfamily of secreted factors comprises more than 30 members including Activins, Nodals, Bone Morphogenetic Proteins (BMPs), and Growth and differentiation factors (GDFs). The TGF-β/activin group includes TGF-β, activin, and Nodal, and the BMP/GDF group includes BMP, GDF, and AMH ligands (Weiss and Attisano, 2013). BMPs govern the intestinal niche homeostasis and balance self-renewal and differentiation (Clevers, 2013; Vermeulen and Snippert, 2014). HNSCC with high baseline BMP-2 protein level are associated with higher rates of local recurrence (Sand et al., 2014). Mulligan et al. indicate that HNSCC CSC upregulate SMURF1 expression to modulate BMP-signaling. This has important implications since non-CSC tumor cells, which display active BMP-signaling, are responsive to current treatments, unlike CSC, which may be more resistant to current therapies and contribute to poor treatment responses. Reactivating BMP-signaling is likely to sensitize CSC to current treatments and improve patient outcomes (Mulligan et al., 2013). Dysregulated TGF-β signaling that functions upstream of Wnt/β-catenin signaling is common in numerous solid tumors, including HNSCC (Bae et al., 2016). The role of TGFβ ligands in HNSCC CSC has not been fully explored. It was demonstrated that TGF-β1 treatment enriches the properties of HNSCC CSC by enhancing sphere formation and increasing self-renewal and stemness-associated gene expression (Oct4 and Sox2) of primary HNSCC CSC. Consecutively, ALDH+ cell population enriched post treatment demonstrating a direct relation between TGF-β signaling and CSC. Following stimulation with TGF-β1, the cells exhibited more resistance to cisplatin and elevated expression of EMT regulators Twist, Snail, and Slug. Bian et al. demonstrated that the loss of TGF-β signaling and PTEN in epithelial cells promotes HNSCC through regulation of both premalignant cells progressing into cancer cells through senescence evasion and the expansion of epithelial stem cells and interactions with the TME in a mouse model. They interpret their findings as significant for the identification of diagnostic biomarkers, as well as for effective treatment strategies targeting both the TGF-β and the PI3K/Akt pathways (Bian et al., 2012). Li K. et al. (2019) showed that TGF-β-induced the activation of AKT rather than ERK1/2 in oral SCC and further illustrated that the non-Smad AKT-FOXO3a axis is essential to regulate the stemness of CSC. There is evidence from the results of different entities such as breast, cervical, and ovarian cancer that the canonical pathway as well as crosstalk of TGF-β are both important factors to regulate the cancer stemness (Chihara et al., 2017; Wu et al., 2017; Matsumoto et al., 2018; Li K. et al., 2019).

The JAK/STAT (Janus kinase/signal transducers and activators of transcription) signaling pathway plays a crucial role in biological processes such as cell proliferation, differentiation, apoptosis, and immune regulation and mediates the cellular response to cytokines. By impairing the pathway, tumor immune surveillance is compromised and tumor formation promoted (Dreesen and Brivanlou, 2007). Besides many other cancer types, aberrant activation of STATs has also been found in HNSCC (Song and Grandis, 2000). JAK/STAT signaling seems to have a functional purpose in survival, self-renewal, and metastasis of CSCs. JAK/STAT signaling in stem cells has been shown to be involved in maintaining embryonic stem cell self-renewal properties, hematopoiesis, and neurogenesis (Chambers, 2004; Stine and Matunis, 2013). In different types of cancer JAK/STAT signaling is aberrantly activated in CSC (Zhou et al., 2007; Birnie et al., 2008). JAK/STAT signaling has been implicated in CSC-mediated metastasis indicating a potential requirement of the pathway for the survival of early metastatic colonies (Calon et al., 2012). In CSC of prostate cancer STAT3 activation by IL-6 or ROS, caused an upregulation of their self-renewal ability (Qu et al., 2013). Growth and survival of CSCs with constitutively activated JAK/STAT were reduced by pharmacologically inhibiting JAK1/2 in an AML model (Cook et al., 2014). As HNSCC is a cancer entity with a high activation level of the JAK/STAT it is most likely that the pathway influences CSC in HNSCC as well. A mechanism which was proposed by Islam et al. (2014) for HNSCC describes that CSC are regulated by RhoC by overexpressing IL-6 and phosphorylation of STAT3. Due to the significance of STAT3 maintaining CSC properties inhibiting the cascade may eliminate CSCs in preventing cancer (Lee et al., 2019).

In humans, the hippo pathway is involved in a multitude of cancer-related physiological and pathophysiological events such as CSC self-renewal, tumorigenesis and anti-cancer drug resistance (Zhao et al., 2011; Moroishi et al., 2015; Zhao and Yang, 2015; Jae Hyung et al., 2018). Expression of genes controlled by the Hippo downstream transcriptional coactivators YAP (Yes-associated protein 1) and TAZ (WWTR1, WW domain containing transcription regulator 1) is widely deregulated in human cancer including HNSCC (Santos-de-Frutos et al., 2019).

Elevated TAZ confers CSC-like properties in diverse cancer contexts (Cordenonsi et al., 2011; Li et al., 2015; Li J. et al., 2019). SOX2 was identified as a direct target of TAZ for the modulation of CSCs self-renewal and maintenance in HNSCC implicating that targeting the TAT/TAZ/TEAD4-SOX2 axis might count as an amenable target for HNSCC therapy (Li J. et al., 2019). Approaches to develop YAP/TAZ inhibitors are currently under investigation (Bae et al., 2016; Shin and Kim, 2020) in the sense of a direct inhibitor between YAP/TAZ and their transcriptional partner TEADs as AP/TAZ signaling might not be as substantial for the normal homeostasis of adult tissues as for neoplastic progression. Another potential strategy is to target the upstream regulators of YAP and TAZ. The molecular program orchestrated by YAP is associated with poor prognosis and tumor progression in HNSCC providing a rationale for developing new therapies targeting the Hippo pathway effector comprising compounds that are already approved for other indications and are currently under preclinical evaluation (Taccioli et al., 2015; Segrelles et al., 2018).

The ErbB family, including EGFR, is known as a key player in cellular processes such as metastasis, tumorigenesis, cell proliferation, and drug resistance, all features related to CSC. It has been shown that EGFR activation increases the expression of stem cell marker CD44. Blocking EGFR tyrosine kinase domains reduces both stem cell maintenance and EMT, and loss of CD44 down-regulates both total and phosphorylated EGFR (Abhold et al., 2012; Perez et al., 2013). These observations suggest that pharmacological blockade of EGFR signal transduction may affect CSC preservation. The effects of cetuximab and erlotinib on CSC were examined in a HNSCC *in vitro* model (Fernanda Setúbal Destro Rodrigues et al., 2018). The drugs caused a decrease in proliferation for all subpopulations and large cellular shifts between the subpopulations EMT-CSC, Epi-CSC and differentiating cell compartments. Loss of EMT-CSCs reduced cell motility and as a consequence presumably a reduction of invasion and metastasis. EGFR inhibition also induced shifts of Epi-CSCs into the differentiating cell compartment which typically is more chemo/radiation-sensitive. This might be a desired effect to enhance the response of tumor cell populations to adjunctive therapies. Abhold et al. also investigated the potential function of EGFR as a regulator of stemness in HNSCC. After *in vitro* activation of EGFR stem cell markers CD44, BMI-1, Oct-4, NANOG, CXCR4, and SDF-1 as well as increased tumorosphere formation were induced, effects that were reversible by administering the EGFR kinase inhibitor Gefinitib. After pharmacological EGFR blockade, the invasion ability was reduced in CSC and the cells showed an increased response toward to cisplatin-induced death. Targeting CSC by blockade of EGFR potentially prevents relapse and secondary tumors and should be taken into account when scheduling HNSCC treatment (Abhold et al., 2012). Another example for a RTK associated with CSC is MET which was shown to be expressed in HNSCC cells having the capacity for self-renewal (Sun and Wang, 2011). Met(+) HNSCC cells built spherical colonies in contrast to MET(-) cells and increasingly expressed self-renewal pathways. The MET receptor was also responsible for cisplatin resistance, tumor formation and metastatic spread in a mouse model. The authors suggested to gain more evidence about the c-Met(+) HNSCC population which might be relevant for tumor progression.

### Targeting CSC Surface Markers

A very specific therapeutic target to point at are CSC surface markers, e.g., CD44 or CD133. Examples for such membrane markers have been introduced above. Specific antibodies targeting these surface markers are currently under investigation. Especially in leukemia, CD44 has been utilized among others to specifically target leukemia stem cells in human AML. In each case, treatment with antibodies against these cell surface markers dramatically decreased malignant potential and eradicated CSCs in mice (Chen et al., 2013). In treating breast cancer, an anti-CD44 antibody-conjugated gold nanorod has been used to target and ablate CD44 positive cells. Using this approach, CD44-targeted cells absorb infrared light resulting in increased local temperature leading to apoptosis (Alkilany et al., 2012). For HNSCC, Su et al. explored a method for preparing anti-CD44 antibody-modified superparamagnetic iron oxide nanoparticles. After co-culturing these with stem cells, the majority of nanoparticles penetrated into the cytoplasm. After alternating magnetic field treatment, the modified nanoparticles induced CSCs to undergo apoptosis. These results demonstrate that CSCs in HNSCC can be eradicated using CD44-targeted magnetic nanoparticles (Su et al., 2019). CD133 was already introduced to play a crucial role in many types of CSCs besides HNSCC, e.g., lung and breast cancer. Treatment with carbon nanotubes conjugated with anti-CD133 monoclonal antibodies followed by radiation with infrared laser light can selectively target CD133 positive glioblastoma cells. Photothermolysis caused by nanotubes specifically eradicates targeted cells (Wang et al., 2011). Kobayashi H and Choyke PL summarized that near-infrared photoimmunotherapy can be applied to any cancer with overexpressed target membrane proteins for which there is a suitable monoclonal antibody. A special remark is made to near-infrared photoimmunotherapy directed at CD44 and CD133, which are not only considered markers of CSCs in breast cancer and glioblastoma but also in HNSCC (Kobayashi and Choyke, 2019).

## Impact of Cancer Stem Cells on Field Cancerization in HNSCC

The “field cancerization” (FC) concept, first described by Slaughter et al. (1953) is based on the observation that normal mucosa adjacent to the tumor contains certain pre-neoplastic genetic fingerprints, which can consecutively cause the development of local recurrence or second primary tumors after abnormal tissue is still residing after surgery (Willenbrink et al., 2020). Normal mucosa adjacent to the tumor acquires molecular alterations in case of carcinogen exposure, i.e., mutations in oncogenes/tumor suppressors, loss of heterozygosity (LOH), and genomic instability. In a multi-step operation, molecular events transform normal epithelium into cancer cells. In theory, only cells that inhabit the epithelia long enough, such as CSC, might accumulate all these genetic alterations. CSC harbor tumor-propagating features and are among the cells accounting for tumor initiation and development. Therefore, it appears practical that CSC might be capable of orchestrating the development of pre-cancerous cells in areas of FC and their transformation into malignant cells (Simple et al., 2015). Furthermore, CSC are considered to have intra-epithelial migratory capability (Biddle et al., 2011). One major observation is the identification of CSC markers in tumor-adjacent normal epithelium supporting the idea of a relation between CSC and FC (Gallmeier et al., 2011; Suresh et al., 2012). Vice versa, molecular FC markers are detected in CSC such as LOH in certain genetic regions like 3p, 0921, 17p11-12, and 13q, most of them encoding for genes that are related to CSC functions. Simple et al. (2015) propose a model of FC orchestrated by CSC. They assume that CSC stem from de-differentiated tumor cells. Another thesis is that normal stem cells (NSC) transform into CSC which is backed by the fact that NSC inhabit the epithelium long enough to collect crucial genetic alterations. The hypothesis of CSC driving FC is substantiated by cancer-related genetic alterations in chromosome positions 3p, 9p, 8p, and 18q. A hit at 13q where Rb gene is situated eventually leads to carcinoma development among subclones that have already transformed from NCS into CSC. In summary, the thesis of an involvement of CSC in FC is sustained by the identification of CSC specific markers in the epithelium adjacent to invasive cancers and the characteristic features of CSC such as tumor-promoting initiation and migration.

## Mathematical Models for Predicting Survival and for CSC Plasticity

Mathematical models are becoming increasingly attractive for capturing data from experimental studies in the wider context of tumor growth dynamics. Emerick et al. (2013) introduced a predictive cancer model and web-based calculator, which estimated the risk of death for HNSCC patients. The binary-biological model integrates data on tumor size, lymph node metastases, and further prognostic factors into estimation of dying from cancer. The model is based on equations such as the relationship between tumor size and the risk of death (the SizeOnly equation) and the relationship between size, number of positive nodes, and the risk of death (the Size 1 Nodes equation). Associations between the risk factors and the actual death risk were precisely captured. Between tumor size and risk of cancer death the risk was monotonically increasing with each cm in tumor size across all HNSCC sublocalizations. Enderling et al. (2019) propose a model for predicting patient-specific RT responses in HNC as a tool for customized radiation dose fractionation. The model aims at the integration of biological differences into clinical RT parameters. In the so-called genomic-adjusted radiation dose (GARD) variables such as the individual patient radiosensitivity index, and the radiation dose or fractionation schedule planned for each patient are implemented. The rationale is to increase dosages in case of resistant cancers and decrease complication risk by lowering the dose in case of sensitive tumors. Although it is acknowledged that other measures of radiosensitivity or biological parameters such as hypoxia or immune responses, and cellular processes including proliferation and DNA repair, are not yet considered, the model is an approach to genomically inform radiation dose (Caudell et al., 2017; Scott et al., 2017).

Stochastic process-based methods have also been developed for modeling growth features and sensitivity of CSC. To undergo quiescence is one feature of stem cells. To analyze how the quiescent state influences treatment, Komarova et al. present a stochastic model to assess how quiescence affects colonic growth before and during CML treatment in the absence of mutations, and how the generation of resistant mutants is influenced by therapy. The model predicted a biphasic response upon therapy, with a fast first phase and a slower second phase. The authors concluded that additional clinical data is needed to determine whether alternative response patterns can also be observed (Komarova, 2006). Hence, mathematical models are valuable to describe how therapy changes the tumor milieu. *In silico* simulation of the influence of an environmental context on the expression of stem-like properties might reveal new insights into the association of stemness with niche-related phenotypic plasticity and add to the development of new treatments. Picco et al. (2017) introduce a mathematical model of context-driven CSC plasticity in which stemness continuously varies across a phenotypic spectrum, directly driven by the environment of breast cancer cells. The hypothesis that all cancer cells are able to adapt to an invasive stem-like phenotype showing the capability of cancer initiation and repopulation. The model calculates main features of stemness varying on a scale from the highest stemness (high invasiveness, self-renewal ability) to full differentiation (poor invasion ability, low proliferative activity). The phenotype is directly modulated be the CSC niche, an environment maintaining cancer cells with a high stem cell like degree phenotype. By the use of a hybrid discrete-continuum (HDC) model, first described by Anderson (Anderson and Chaplain, 1998; Anderson et al., 2000) cancer cell phenotypes are defined by their grade of stemness. The model calculates the plasticity of cancer cells by their shifting ability on the scale of differentiation, represented by a degree of differentiation, D^*i*^. D^0^ stands for highest stemness, and D^*N*^ stands for full differentiation of a cancer cell with poor stemness. N is the total amount of degrees on the scale. Corresponding to each D^*i*^ is a specific phenotype that determines the remodeling of the ECM (production and degradation rate) by the respective cell and its proliferation capacity. It is likely that the HDC approach could be transferred from breast cancer to other entities. Although the possibilities of mathematical modeling are manifold, one might take into account that tumorigenesis is a multifactorial process. In a recent review it is discussed that most deterministic models show limitations to predict the last phases of cancer growth and cannot predict long-term tumor growth rate (Tabassum et al., 2019). Brady and Enderling critically discuss the fact that mathematical tumor models frequently lack access to high-resolution cancer biology or oncology data including independent training and validation data sets. The models are limited to being merely academic and not feasible to speculate on optimal therapy. It is mandatory to set up a standard to adequately train and validate the models. The purpose of such models should be up-front subclassified into either “academic” or “translational” to prevent loss of credibility (Brady and Enderling, 2019).

## Tumor Organ-On-Chip Models

Current innovations in microfluidic cell culture technology have led to the generation of human organs-on-chips (or organ chips) that mimic cancer cell behavior within a functional tissue and organ-specific context (Bhatia and Ingber, 2014; Skardal et al., 2017; Aleman and Skardal, 2019; Sontheimer-Phelps et al., 2019; Trujillo-de Santiago et al., 2019). These “organs-on-chips” permit the development of novel *in vitro* disease models, and could eventually help to replace animal models for drug development and toxin testing (Huh et al., 2011). The technology allows to examine vascular perfusion and the effects of medication or microenvironmental factors on cancer cells, either alone or enriched with other cell types as epithelial and endothelial cells, fibroblasts, immune cells, then visualizing sensitivity and response in real-time (Sontheimer-Phelps et al., 2019). Another advantage of microfluidic systems is that they offer perfusion causing continuous nutrient supply and waste removal, in turn maintaining a more stable culture environment, and will potentially allow to identify physiochemical mediators of mass transport in the tumor ECM (Avendano et al., 2019).

Technical challenges are the separation and isolation of cancer and stromal cells from the individual patient. The cells have to be re-arranged on organ-chips in the appropriate proportions and location to accurately mimic cell-specific features, thereby considering culture condition and isolation protocols for all different cellular subgroups. The technique is supposed to be elaborate and time-consuming and has to be correlated with existing patient outcome and response data before transferred into the clinical applicability. Another potential limitation is the absorption of small molecules such as certain drugs by the most commonly used material for organ chips, PDMS, so the development of further new materials for organ-chips might be required (Sontheimer-Phelps et al., 2019).

In a recent review the application of tumor-on-chip systems in precision or personalized medicine is addressed (Trujillo-de Santiago et al., 2019). The maintenance of vital tumor tissue from biopsies in microfluidic systems, is described as feasible in different cancer types, including HNSCC (Carr et al., 2014; Astolfi et al., 2016). Kennedy et al. describe a robust, easy to use, tumor-on-a-chip platform, which maintains vital HNSCC samples for the purpose of *ex vivo* irradiation. The model mimicked microvascular flow and diffusion by the use of sintered discs to separate the tissue from media. Viability was sustained for 68 hrs. Tumor biopsies showed an adequate response to fractionated IR. The addition of concurrent cisplatin considerably increased the apoptotic cell fraction. The novel platform is described as cost-effective and transferable to other cancer types (Kennedy et al., 2019).

Some recent papers have described the maintenance of portions of real tumorous tissue from biopsies in microfluidic systems (Trujillo-de Santiago et al., 2019). Bower et al. (2017) were able to sustain the tissue integrity of HNSCC samples for 48 h in a simple microfluidic chamber. Sylvester et al. cultivated HNSCC tumor biopsies in a microfluidic device for up to 9 days after surgical removal. The response of HNSCC tissue samples (fresh and fresh-frozen) to standard anticancer drugs (cisplatin, 5-flurouracil or docetaxel) was monitored. Proliferation and showing similar viability and metabolic cell death were statistically similar in frozen and fresh biopsies. Dose-dependent cell deaths seen after administration of all drugs. Cytotoxicity was enhanced by combination of drugs. The results demonstrate that tumor tissue can be cryogenically stored and analyzed at a later (Sylvester et al., 2013). Although highly sophisticated tumor-on-chip systems are not in any event cost-efficient, it is highly likely that its embodiments will be of translational significance. The assessment of responsiveness of individual tumors by using microfluidic cultures and selection of specific therapy schemes could contribute to new insights into HNSCC biology, to customized care and consecutively to improved prognosis.

## Usage of Biomaterials as Scaffolds for 3D Culture Models

As presented before, the development of novel 3D cultures is based on a better understanding of TME structure and interactions. To improve cellular functions, various forms of biomaterials are already available serving as a scaffold for 3D cell culture which are comprehensively summarized in recent reviews (Gu and Mooney, 2016; Kamatar et al., 2020; Park et al., 2021). One method is the usage of hydrogels, which can be further divided into synthetic and natural. Another cell-culturing technique is based on solid, namely porous and fibrous scaffolds, which is one of the older techniques in the field (Cheng and Kisaalita, 2010). These scaffolds mainly consist of porous foams or fibrous meshes made from synthetic polymers. Decellularized native tissue preserves the natural environment and recapitulates key ECM components, a major advantage compared with artificial scaffolds. The technique is currently under investigation for advanced bioprocessing into organotypic 3D solid tumor models (Ferreira et al., 2020) and there are trends to derive bioinks from decellularized ECM (Dzobo et al., 2019). The surfaces of ultra-low adherent (ULA) plates are coated with polymers enabling spheroid formation of cells as attachment to the surface of the plates is impaired. For instance, ULA plates have been used for testing anti-CSC compounds in the stem cell-enriched HNSCC tumor model by Goričan et al. (2020).

## 3D Bioprinting Models

In addition to the above mentioned methods, a technique developed in the field of tissue engineering emerged in the field of constructing tumor models in the last decade. The development of 3D bioprinting technologies in the biomedical field enabled the construction of artificial tissues with complex structures and various components using different cell types and natural (e.g., collagen, fibrin, etc.) or synthetic (e.g., polyethylene glycol, gelatin-methacrylate, etc.) hydrogels. It is expected that the 3D bioprinting technique will overcome disadvantages of currently existing tumor models like the lack of controllable spatial distribution of tumor cells and ECM compositions (Zhang et al., 2016; Ma et al., 2020). The technical possibilities of 3D bioprinting will allow to arrange different cell types (e.g., cancer cells, CSCs, endothelial cells, CAFs, etc.) and ECM-based biomaterials to generate tumor models that recapitulate the *in vivo* tumor very closely including the architecture of a tumor (Penfornis et al., 2017; Langer et al., 2019) (Figure 1). It is expected that 3D bioprinting will pave the way to both, to reliable models to predict the optimal treatment of a cancer patient and to more reliable models for basic and applied cancer research. Currently, protocols for bioprinted tumor models of glioma, liver, breast, ovarian, and cervical cancers are published as mentioned in the review of Ma et al. (2020). Regarding HNCs, in 2018 a study was published by Almela et al. (2018), in which a 3D printed bone mimicking scaffold was used to investigate the bone invasion of oral squamous cancer cells to develop a cancerous bone oral mucosal model. A protocol to create a bioprinted head and neck tumor model has yet not been published. However, since 3D bioprinting has only emerged in the last decade in the field of constructing tumor models, the authors of this review are convinced that it will become a powerful technique to develop various head and neck tumor models.

## Electrospun Nanofibres

Electrospinning is a well-known process to produce fibrous and porous three-dimensional (3D) materials starting from a polymeric solution. This process was first used in the field of tissue engineering but meanwhile it is also applied to engineer 3D cancer models. These fibrous materials are able to mimic the ECM of living tissue (Cavo et al., 2020). The use of different polymers enables the possibility to adapt the properties of the scaffold to the varying properties of ECMs from different tumor entities. A summary of materials used can be found in the review by Chen S. et al. (2018) in which advantages and disadvantages of this technique are also discussed (Chen S. et al., 2018). Such fibrous scaffolds were developed for many tumor types like breast cancer, pancreatic cancer and many more. A comprehensive summary of tumor types for which such models exist can be found in Cavo’s review (Cavo et al., 2020). However, such models are currently not published for HNC.

## Soft Lithography and Bioimprints

The soft lithography technique is used to produce so-called bioimprints. These bioimprints provide *in vitro* substrates with cell-like features and enables to investigate effects of physical topographies that are similar to those experienced by cells *in vivo* (Tan et al., 2015). Materials used for bioimprints are for example polydimethylsiloxane (PDMS), polystyrene (pST), polymethacrylate (pMA), and some others (Murray et al., 2014; Mutreja et al., 2015; Tan et al., 2015). How such bioimprints are created is well described in the studies of Murray et al. (2014), Tan et al. (2015).

One limitation is that both of the above mentioned techniques lack mimicking the complete microenvironment, including vascularization and immune cells (Chen S. et al., 2018). For HNC cells, this method has not yet been established.

## Future Challenges

**FIGURE 2:**
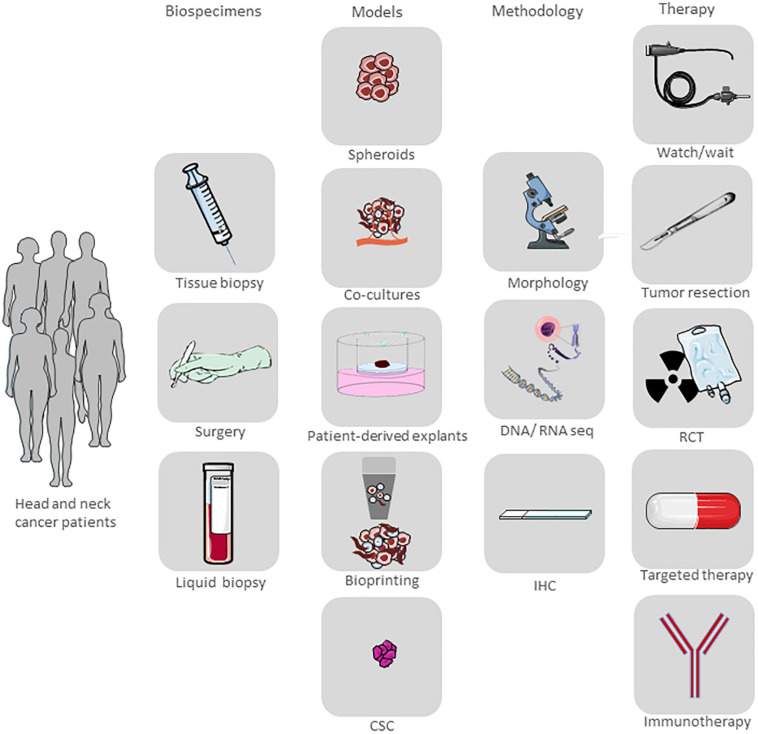
Vision of a translational HNSCC platform. The platform ultimately aims to shorten time to diagnosis and treatment, predict tumor relapse or progress and identify targetable mutations after treatment by analysis of different biospecimen with established and new, innovative technologies. Overall, developing a precision medicine platform for head and neck malignancies will improve survival in patients with a dismal clinical outcome. Parts of the figure were drawn by using pictures from Servier Medical Art (http://smart.servier.com/), licensed under a Creative Commons Attribution 3.0 Unported License (https://creativecommons.org/licenses/by/3.0/).

As summarized in this review article, clinical implementation of chemoresponse assays and biomarkers as well as therapeutic targets will enable patient stratification based on molecular characterization of the tumor and TME by 3D modeling (Figure 2). In HNSCC, however, reliable models that are predictive of clinical efficacy remain scarce. So far, no description of a successful translation of chemosensitivity assays or predictive models into the clinical routine has been published for HNSCC. Druggable target components in HNSCC have been identified and more are being discovered due to the development of novel technologies and acquisition of competence in the field of personalized medicine. Despite all these innovations specific tumor-related challenges need to be considered. Due to the frequent development of therapy resistance there is an absolute need to unveil the underlying mechanisms in order to develop strategies for circumvention. Although it has been demonstrated that combinatorial regimens might be advantageous there are still no standardized tumor models available that allow predicting the efficacy of these combinations and testing the individual sensitivity of the tumor to be able to select from an abundance of therapeutic agents. After having identified a suitable therapeutic approach, the tumor ideally should be constantly monitored to discover the outgrowth of resistant clones as early as possible. Also, further characterization of the architecture and physiology of CSC-enriched spheroids in future studies will be crucial for future therapy development.

Optimizing current therapies and developing new therapeutic targets is an ambitious aim in HNSCC research in order to improve its prognosis. The further establishment of suitable 3D cancer models will be an essential in this process.

## Author Contributions

AA, JK, and NR conceived the topic for this review. All authors listed have made a substantial, direct and intellectual contribution to the work, and approved it for publication.

## Conflict of Interest

The authors declare that the research was conducted in the absence of any commercial or financial relationships that could be construed as a potential conflict of interest.
